# Gynecological issues in children and adolescents seen at rare-disease referral centers: an observational retrospective cohort study

**DOI:** 10.1186/s13023-025-03618-2

**Published:** 2025-03-11

**Authors:** Iphigénie Cavadias, Magali Viaud, Marie Falampin, Alaa Cheikhelard, Karinne Gueniche, Chloé Ouallouche, Dinane Samara-Boustani, Damien Bonnet, Nadia Bahi-Buisson, Pierre Quartier-dit-Maire, Smaïl Hadj-Rabia, Laurence Heidet, Slimane Allali, Pascale de Lonlay, Jeanne Amiel, Rima Nabbout, Despina Moshous, Valérie Cormier-Daire, Arnaud Picard, Isabelle Desguerre, Isabelle Sermet-Gaudelus, Graziella Pinto, Dominique Bremond-Gignac, Frank Ruemmele, Muriel Girard, Véronique Abadie, Syril James, Annie Harroche, Michel Polak, Sabrina Da Costa

**Affiliations:** 1 Pediatric Endocrinology, Diabetology, Gynecology Department, Necker-Enfants Malades University Hospital, AP-HP Centre, 75015 Paris, France; 2 Gynecology Department, Port-Royal University Hospital, AP-HP Centre, 75014 Paris, France; 3 Faculty of Medicine, Université Paris Cité, 75006 Paris, France; 4 Reference Center for Rare Gynecological Pathologies (PGR), Pediatric Endocrinology, Diabetology, Gynecology Department, Necker-Enfants Malades University Hospital, AP-HP Centre, 75015 Paris, France; 5 Paediatric Surgery and Urology Department, Necker-Enfants Malades University Hospital, AP-HP Centre, 75015 Paris, France; 6 Reference Center for Rare Growth and Development Endocrine Diseases (CMERC), Necker-Enfants Malades University Hospital, AP-HP Centre, 75015 Paris, France; 7 Reference Center for Rare Diseases of Pituitary Origin (HYPO), Necker-Enfants Malades University Hospital, AP-HP Centre, 75015 Paris, France; 8 M3C-Necker, Necker-Enfants Malades University Hospital, AP-HP Centre, 75015 Paris, France; 9 Reference Center for Intellectual Disabilities of Rare Causes (DICR), Necker-Enfants Malades University Hospital, AP-HP Centre, 75015 Paris, France; 10 Reference Center for Inflammatory Rheumatic Diseases, Autoimmune Diseases and Systemic Interferonopathies in Children (RAISE), Necker-Enfants Malades University Hospital, AP-HP Centre, 75015 Paris, France; 11 Reference Centre for Genodermatoses and Rare Skin Diseases (MAGEC), Necker-Enfants Malades University Hospital, AP-HP Centre, 75015 Paris, France; 12 Reference Center for Renal Hereditary Disease for Children and Adults (MARHEA), Necker-Enfants Malades University Hospital, AP-HP Centre, 75015 Paris, France; 13 Reference Centre for Sickle Cell Disease, Necker-Enfants Malades University Hospital, AP-HP Centre, 75015 Paris, France; 14 Reference Center for Inherited Metabolic Diseases in Children and Adults (MAMEA), Necker-Enfants Malades University Hospital, AP-HP Centre, 75015 Paris, France; 15 Reference Center for Developmental Anomalies and Malformative Syndromes of Île-de-France (Genetics), Necker-Enfants Malades University Hospital, AP-HP Centre, 75015 Paris, France; 16 Reference Center for Rare Epilepsies (CRéER), Necker-Enfants Malades University Hospital, AP-HP Centre, 75015 Paris, France; 17 Reference Center for Primary Immunodeficiencies (CEREDIH), Necker-Enfants Malades University Hospital, AP-HP Centre, 75015 Paris, France; 18 Reference Center for Constitutional Bone Diseases (MOC), Necker-Enfants Malades University Hospital, AP-HP Centre, 75015 Paris, France; 19 Reference Center for Clefts and Facial Malformations (MAFACE), Necker-Enfants Malades University Hospital, AP-HP Centre, 75015 Paris, France; 20 Reference Center for Neuromuscular Diseases, Necker-Enfants Malades University Hospital, AP-HP Centre, 75015 Paris, France; 21 Reference Center for Cystic Fibrosis and Conditions Linked to a CFTR Abnormality, Necker-Enfants Malades University Hospital, AP-HP Centre, 75015 Paris, France; 22 Reference Center for Prader-Willi Syndrome and Other Rare Forms of Obesity With Eating Disorders, Necker-Enfants Malades University Hospital, AP-HP Centre, 75015 Paris, France; 23 Reference Center for Rare Eye Diseases (OPHTARA), Necker-Enfants Malades University Hospital, AP-HP Centre, 75015 Paris, France; 24 Reference Center for Rare Digestive Diseases (MaRDI), Necker-Enfants Malades University Hospital, AP-HP Centre, 75015 Paris, France; 25 Reference Center for Biliary Atresia and Genetic Cholestases in Children (CRAVB-CG), Necker-Enfants Malades University Hospital, AP-HP Centre, 75015 Paris, France; 26 Reference Center for Pierre Robin Syndrome and Congenital Sucking-Swallowing Disorders (SPRATON), Necker-Enfants Malades University Hospital, AP-HP Centre, 75015 Paris, France; 27 Reference Center for Chiari and Vertebral and Spinal Malformations - Spinal Dysraphism (C-MAVEM), Necker-Enfants Malades University Hospital, AP-HP Centre, 75015 Paris, France; 28 Reference Center for Constitutional Hemorrhagic Diseases, Necker-Enfants Malades University Hospital, AP-HP Centre, 75015 Paris, France; 29 Cochin Institute, INSERM U1016, 75014 Paris, France; 30 IMAGINE Institute Affiliate, INSERM U1163, 75015 Paris, France

**Keywords:** Cohort, Rare disease, Gynecology, French National Referral Center

## Abstract

**Background:**

The current development of gynecology services for children and adolescents seeks to meet needs both in the overall population and in patients with rare diseases. In France, the referral center for rare gynecological diseases specializes in four major types of conditions, namely, uterovaginal malformations, hereditary hemorrhagic diseases, rare benign breast diseases, and gynecological repercussions of rare chronic diseases.

**Objective:**

To describe consecutive patients who had a first visit in 2018–2023 at the referral center for rare gynecological diseases at the Necker Pediatric University Hospital in Paris, France, and who were diagnosed with a condition in any of the four categories listed above.

**Material and methods:**

For this single-center retrospective observational cohort study, data from the referral-center database were collected and reviewed. These data included year of birth, age at and reason for first gynecology visit, and rare chronic disease and referring rare-disease center for patients seen for gynecological repercussions of rare chronic diseases.

**Results:**

The 704 included patients had a median age of 15.2 years (interquartile range 3.8) at the first visit. Among them, 100 (14.2%) had uterovaginal malformations, 32 (4.6%) hereditary hemorrhagic diseases, 17 (2.4%) rare benign breast diseases, and 555 (78.8%) gynecological repercussions of rare chronic diseases. The leading reasons for the visit were dysmenorrhea (15.6%), menorrhagia (15.5%), uterovaginal malformations (15.2%), and irregular periods (14.9%).

**Conclusion:**

Repercussions of rare chronic diseases managed at rare-disease referral centers were by far the leading reason for seeking gynecological expertise in rare diseases. In this complex situation, the underlying disease and its treatments interact with the gynecological manifestations and their treatment, requiring close collaboration among all specialists caring for each patient.

## Background

Pediatric and adolescent gynecology is being developed to meet the increasingly recognized needs of these young patients [1, 2]. Puberty disorders, dysmenorrhea, menorrhagia, and irregular menstrual cycles are the main symptoms. Rare gynecological diseases exist, and several non-gynecological rare chronic diseases can produce gynecological manifestations. In France, referral centers for rare diseases were created starting in 2004 [3] to decrease diagnostic and therapeutic delays and to improve quality of care. All these centers record data prospectively into a national database (CEMARA initially [4] then BaMaRa starting in 2017) using standardized data-collection methods, with the aim of improving our understanding of rare diseases and facilitating research [5, 6]. The referral center for rare gynecological diseases specializes in the management of uterovaginal malformations, hereditary hemorrhagic diseases, rare benign breast diseases, and gynecological repercussions of rare chronic diseases.

The aim of this retrospective observational cohort study was describing the characteristics of patients who had a first visit at the gynecology clinic of the Necker referral center for rare gynecological diseases in 2018–2023. Data from the referral-center database were systematically analyzed.

## Material and methods

The study was approved by the appropriate ethics committee (CEERB) on July 10 2024 (N°2024 0710162635). The BaMaRa database used as the data source is registered with the French data protection agency (CNIL). In accordance with French law, patient consent to inclusion in the database and to use of de-identified data for research was obtained before data collection was started.

### Population

Consecutive patients seen for the first time at the gynecology clinic of the referral center at the Necker Pediatric University Hospital (Paris, France) between November 2018 and May 2023 were eligible for this observational cohort study. The inclusion criteria were a diagnosis of uterovaginal malformation, hereditary hemorrhagic disease, rare benign breast disease, or gynecological repercussions of a rare chronic disease.

### Data collection and patient classification

The BaMaRa database includes clinical information extracted from medical records of all patients diagnosed with any of the four categories of rare gynecological issues. These data include year of birth, age at the first referral-center visit, reason for the first visit, and, for patients with rare chronic diseases, nature of the disease and referring rare-disease center.

Patients were divided into four groups according to whether the diagnosis was a uterovaginal malformation, a hereditary hemorrhagic disease, a rare benign breast disease, or gynecological repercussions of a rare chronic disease. This last group was further classified into sub-groups based on the referring center, i.e., on the underlying rare chronic disease, as listed in Table 1. For patients receiving follow-up at more than one rare-disease center, the center that referred the patient to the Necker gynecological referral center was used for the statistical analysis.

**Table 1:** Categories of pediatric chronic rare diseases with the corresponding referral centers in France

Rare diseases	Referral center
Biliary atresia and genetic cholestasis	AVB-CG
Chiari and vertebral and spinal malformations—Chiari and syringomyelia	C-MAVEM
Primary Immunodeficiencies	CEREDIH
Mast-cell disorders	CEREMAST
Rare vascular diseases of the central nervous system and retina	CERVCO
Rare endocrine diseases affecting growth and development	CMERC
Rare epilepsies	CRéER
Rare adrenal**-**gland diseases	CRMRS
Vascular liver diseases	CRMVF
Cystic fibrosis and conditions linked to a CFTR abnormality	Cystic fibrosis
Toxic bullous dermatoses and severe toxidermia	DBTTG
Intellectual disabilities due to rare causes	DICR
Genetic hearing loss	Genetic Deafness
Developmental anomalies and malformation syndromes	Genetics
Rare diseases of pituitary origin	HYPO
Clefts and other facial malformations	MAFACE
Rare genetic diseases of the skin and mucous membranes	MAGEC
Rare ENT malformations	MALO
Inherited metabolic diseases	MAMEA
Rare digestive diseases	MaRDI
Hereditary kidney diseases	MARHEA
Neuro-inflammatory diseases	MIRCEM
Constitutional bone diseases	MOC
Complex congenital heart defects	M3C
Neuromuscular diseases	Neuromuscular diseases
Rare ophthalmological disorders	OPHTARA
Rare gynecological disorders	PGR
Prader-Willi syndrome and other rare forms of obesity with eating disorders	PRADORT
Rare insulin-secretion and insulin-sensitivity disorders	PRISIS
Inflammatory joint diseases and rare systemic autoimmune diseases	RAISE
Rare respiratory diseases	RESPIRARE
Major sickle-cell syndromes and other rare disorders of erythrocytes and erythropoiesis	Sickle cell
Pierre Robin syndrome and congenital impairments of sucking/swallowing	SPRATON

*CFTR* cystic fibrosis transmembrane conductance regulator, *ENT* ear, nose, and throat

### Statistical analysis

Continuous variables are described as median (interquartile range) or mean (standard deviation) and categorical variables as n (%). Means are compared using a statistical test based on a one-way analysis of variance. The validity conditions of the one-way analysis of variance were verified: independence of observations, identical variance of the studied variable in each of the compared subgroups, and normality of the model's residuals. The normality of the model's residuals was visually verified using a diagram. A p-value less than 0.05 was considered statistically significant. The analysis was done using the R program, version 4.1.1 (https://www.r-project.org).

## Results

**Fig. 1:**
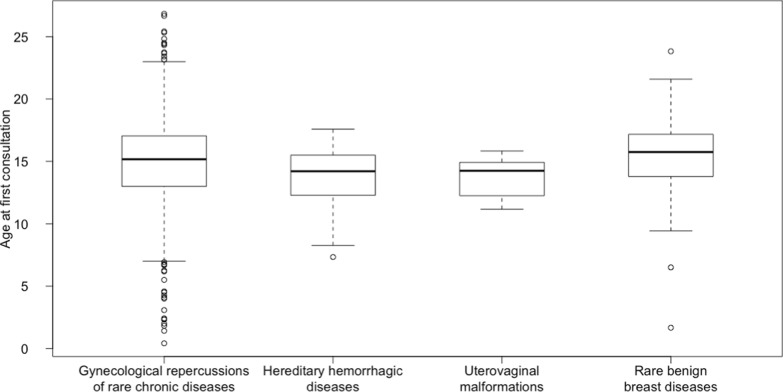
Median age at the first gynecology visit according to diagnostic sub-group. Each box indicates the interquartile range, the horizontal line in the box indicates the median, and the whiskers show the range of the data, excluding outliers

**Fig. 2:**
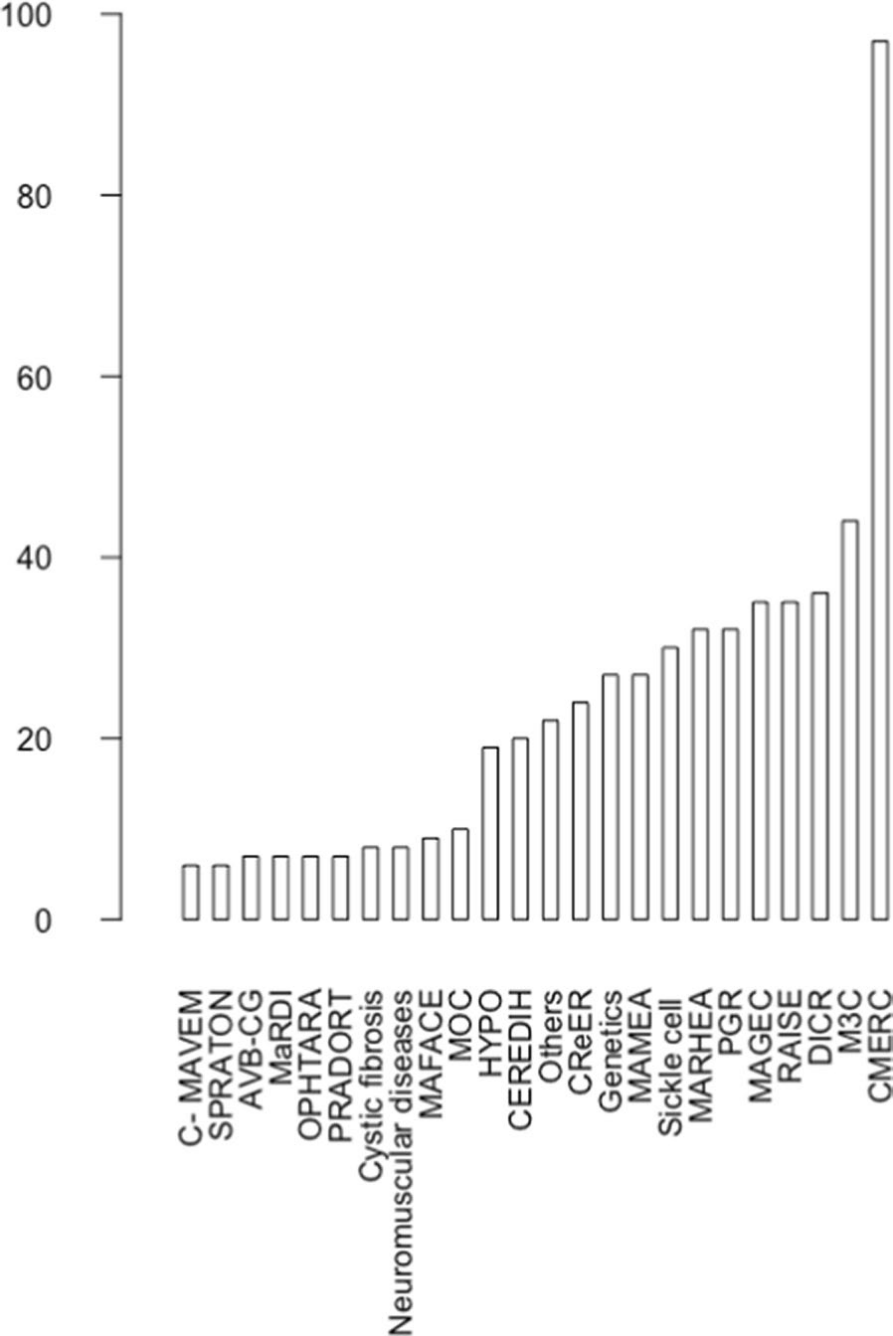
Number of patients in each rare-disease category. Of the 704 patients included in the study, 555 had rare chronic diseases. The names of the rare-disease centers are on the X axis and the corresponding diagnoses are shown in Table 1. The PGR is the Necker referral center for rare gynecological diseases. All other patients were referred to the PGR by other rare-disease centers. The main diagnoses in the patients not referred by other rare-disease centers (n = 32) were premature ovarian failure, congenital hypogonadotropic hypogonadism, and immature ovarian teratomas. The bar labeled “other” indicates the patients referred by any of the nine rare-disease centers that each referred fewer than 5 patients to the gynecological rare-diseases center during the study period (RESPIRARE, MALO, CERVCO, CEREMAST, Genetic deafness, PRISIS, MIRCEM, CRMVF, DBTTG; see Table 1)

Seven hundred and four patients who had their first visit between November 2018 and May 2023 at the gynecology clinic of the Necker referral center for rare gynecological diseases were included in the study. The diagnosis was uterovaginal malformation in 100 (14.2%) patients, hereditary hemorrhagic disease in 32 (4.6%) patients, rare benign breast disease in 17 (2.4%) patients, and gynecological repercussions of a rare chronic disease in 555 (78.8%) patients. Table 2 lists the reasons for the first visit in the study population. Median age at the first visit was 15.2 years (interquartile range 3.8) (Fig. 1). There was no significant difference in age at the first visit between the four diagnostic groups (p = 0.141). Figure 2 shows the distribution of the 555 patients with gynecological repercussions of rare chronic diseases according to the referring rare-disease center. Table 3 lists the reasons for the first visit and age at first visit in the sub-group of patients with rare chronic diseases. There was no significant difference in age at the first visit between the sub-groups of patients with rare chronic diseases (p = 0.176). About 33% of patients with endocrine disorders affecting growth and development were referred for pubertal induction. Menorrhagia and dysmenorrhea were the most common reasons for referral by the center for complex congenital heart diseases (respectively 40.9% and 31.8% of patients). The main reasons for referral among patients with intellectual disabilities were premature puberty (19.4%) and menstrual suppression to facilitate care (16.7%). Dysmenorrhea was the main reason in patients with inflammatory and auto-immune diseases (31.4%), hereditary kidney diseases (25%) and sickle-cell syndromes (53.3%).

**Table 2:** Reasons for first visit to the referral center for rare gynecological diseases

Reason for first visit	N patients
Dysmenorrhea	110
Menorrhagia	109
Uterovaginal malformation	107
Irregular periods	105
Gynecological advice^a^	89
Pubertal induction	75
Contraception	42
Early puberty	32
Breast disease	28
Menstrual suppression	21
Clinical hyperandrogenism	15
Premenstrual syndrome	10
Ovarian cyst	4
Hyperprolactinemia	4

The total is not 704 because some patients had more than one reason for first visit

^a^in some cases for patients requiring only one-off advice with no follow-up visits

**Table 3:** Age at first visit and reasons in patients with rare chronic diseases

	CMERC N = 97	M3C N = 44	DICR N = 36	MAGEC N = 35	RAISE N = 35	PGR N = 32	MARHEA N = 32	Sickle cell N = 30	MAMEA N = 27	Genetics N = 27	CRéER N = 24	CEREDIH N = 20	HYPO N = 19
*Reasons for first visit* *N (%)*													
Premenstrual syndrome	1 (1)	2 (4.5)	1 (2.8)	2 (5.7)	0 (0)	0 (0)	0 (0)	0 (0)	0 (0)	1 (3.7)	2 (8.3)	0 (0)	0 (0)
Ovarian cyst	0 (0)	0 (0)	0 (0)	1 (2.9)	0 (0)	3 (9.4)	0 (0)	0 (0)	0 (0)	0 (0)	0 (0)	0 (0)	0 (0)
Breast disease	0 (0)	1 (2.3)	2 (5.6)	3 (8.6)	1 (2.9)	0 (0)	0 (0)	1 (3.3)	1 (3.7)	0 (0)	0 (0)	0 (0)	0 (0)
Hyperprolactinemia	1 (1)	0 (0)	1 (2.8)	1 (2.9)	0 (0)	0 (0)	0 (0)	0 (0)	1 (3.7)	0 (0)	0 (0)	0 (0)	0 (0)
Menstrual suppression	0 (0)	1 (2.3)	6 (16.7)	1 (2.9)	1 (2.9)	0 (0)	0 (0)	0 (0)	1 (3.7)	4 (14.8)	**5 (20.8)**	0 (0)	0 (0)
Clinical hyperandrogenism	4 (4.1)	0 (0)	0 (0)	1 (2.9)	1 (2.9)	0 (0)	1 (3.1)	0 (0)	0 (0)	2 (7.4)	1 (4.2)	0 (0)	0 (0)
Menorrhagia	7 (7.2)	**18 (40.9)**	4 (11.1)	4 (11.4)	7 (20)	1 (3.1)	6 (18.8)	1 (3.3)	**7 (25.9)**	3 (11.1)	4 (16.7)	0 (0)	3 (15.8)
Pubertal induction	**32 (33)**	1 (2.3)	1 (2.8)	0 (0)	1 (2.9)	**11 (34.4)**	0 (0)	4 (13.3)	2 (7.4)	2 (7.4)	0 (0)	**10 (50)**	**8 (42.1)**
Early puberty	2 (2.1)	0 (0)	**7 (19.4)**	4 (11.4)	0 (0)	0 (0)	2 (6.2)	1 (3.3)	2 (7.4)	3 (11.1)	2 (8.3)	0 (0)	1 (5.3)
Dysmenorrhea	5 (5.2)	14 (31.8)	5 (13.9)	4 (11.4)	**11 (31.4)**	1 (3.1)	**8 (25)**	**16 (53.3)**	4 (14.8)	4 (14.8)	4 (16.7)	2 (10)	2 (10.5)
Contraception	6 (6.2)	6 (13.6)	1 (2.8)	4 (11.4)	4 (11.4)	0 (0)	3 (9.4)	5 (16.7)	1 (3.7)	1 (3.7)	3 (12.5)	0 (0)	0 (0)
Uterovaginal malformation	0 (0)	0 (0)	0 (0)	3 (8.6)	0 (0)	2 (6.2)	0 (0)	0 (0)	0 (0)	0 (0)	0 (0)	0 (0)	0 (0)
Gynecological advice	24 (24.7)	4 (9.1)	5 (13.9)	**7 (20)**	3 (8.6)	4 (12.5)	**8 (25)**	2 (6.7)	6 (22.2)	3 (11.1)	4 (16.7)	1 (5)	0 (0)
Irregular periods	18 (18.6)	3 (6.8)	3 (8.3)	3 (8.6)	8 (22.9)	**11 (34.4)**	7 (21.9)	3 (10)	4 (14.8)	**6 (22.2)**	0 (0)	7 (35)	7 (36.8)
*Age at first visit* *Mean (standard deviation)*													
	15.1 (4.1)	15.6 (2.2)	14.2 (4.3)	13.1 (3.8)	15.5 (2.2)	14.1 (3.6)	14.0 (4.6)	16.8 (3.4)	15.0 (4.5)	14.0 (5.3)	14.2 (3.8)	14.7 (2.0)	15.1 (3.1)

^a^For the correspondence between center names and diagnoses, see Table 1

^b^Only the centers that referred at least 10 patients are considered: the total number of patients is 458

^c^For each diagnostic category (rare-disease center), the most common reason is in bold type

## Discussion

This study describes issues and profiles of patients seen for the first time at a rare gynecological-disease referral center in a Pediatric University Hospital. Between November 2018 and May 2023, 704 patients were diagnosed with rare gynecological conditions. Median age at the first gynecological visit was about 15 years, consistent with the frequent link of symptoms with menstruation. At this age, care by adult gynecologists would not be appropriate [7]. Dysmenorrhea, menorrhagia, and irregular menstrual cycles were common reasons for the first visit. In a previous study, these symptoms were also frequent in females aged 15–25 years who did not have rare diseases [8].

Most patients had underlying rare chronic diseases of childhood or adolescence requiring specific expertise in the pediatric age group. The disease itself and/or its treatment may result in gynecological manifestations and, in turn, these manifestations or hormone therapy given to treat them may affect the underlying disease. Rare diseases or their treatments may be associated with premature or delayed puberty. Dysmenorrhea may trigger or worsen the manifestations of the underlying disease, for instance epileptic seizures or vaso-occlusive crises in sickle-cell disease. Menorrhagia may exacerbate underlying anemia in patients on long-term anticoagulant therapy. Contraception may be required in patients receiving teratogenic drugs to treat rare chronic diseases. In-depth knowledge of possible interactions between treatments and rare chronic diseases is essential. Thus, enzyme-inducing drugs may modify the effects of hormonal treatments, and hormonal treatments may modify the effects of medications given for rare chronic diseases.

The small proportion of patients seeking contraception is consistent with the young age. It is important for the physician to know the contraindications to certain contraceptives related to the chronic pathology or the drug interactions between contraceptives and the basic treatments of the chronic disease. The implementation of contraception must subsequently be re-evaluated in the following months to assess tolerance and make adjustments if necessary. It is essential to find the contraception that best suits the patients to improve adherence. Even in the absence of a contraceptive need, it is essential that these young girls and women are aware of the impact of a possible pregnancy on the balance of their chronic pathology and the interest of planning a pregnancy with specialist physicians.

During the first gynecological visit, patients are interviewed to allow the detection of previously overlooked symptoms. Menorrhagia, for instance, is often an underestimated symptom by families and little sought by healthcare providers. Very heavy first menstrual cycles are sometimes the entry point into the bleeding disease in young girls. It is important that pediatricians are sensitized and think to ask patients about the age of onset of their first menstrual cycles, their regularity, their duration, their abundance, assessable for example by the Higham score [9], and associated symptoms such as pain, given that they are the main interlocutors of these young patients. The Higham score is one of the most objective ways to quantify the abundance of menstrual cycles and identify heavy menstrual cycles. Patients count the number of protections used per day specifying with the pictogram if they are lightly filled (1 point per change), moderately filled (5 points per change), or abundantly filled (20 points per change) specifying the presence of clots and overflow. A score evaluated at 100 over the menstrual cycle corresponds to a bleeding of 80 ml and thus to menorrhagia.

During follow-up at the gynecology referral center, patients are given ample opportunities to receive information about contraception, the prevention of and screening for sexually transmitted diseases, consent, other issues related to sexuality and the human papillomavirus (HPV) vaccination. In France, HPV vaccination is recommended between the 11 and 14 years of age and catch-up immunization is offered between 15 and 19 years of age [10]. The future of the patients as adult women and possible fertility issues are also discussed.

The previous considerations demonstrate that specific gynecological expertise is essential for patients with rare chronic diseases. The opinion of the referral center for rare gynecological diseases can be sought either occasionally or for long-term follow-up depending on the complexity of the situation. It is advisable to refer to a referral center due to the rarity of these diseases and the constant evolution of research and knowledge about the diseases. Centralizing data would also allow for larger cohorts to advance knowledge in each type of pathology to enable better management. For patients with uterovaginal malformations, there is no indication for specific management in childhood and adolescence in the absence of symptoms. The issue may arise at an older age at the start of sexual activity or at the time of pregnancy desire. The opinion of the referral center is sometimes sought late after unnecessary surgeries probably due to physicians' lack of knowledge of specific management. However, it is important to manage these patients in referral centers so that they receive appropriate information and often inherent psychological support. For patients with rare breast diseases, discovery is mostly following symptoms, and management is either through hormonal medical treatments or surgeries. Due to the rarity of these diseases and the more frequent risks of recurrence, it is essential to have a specialized opinion in order to offer the best possible management and ensure follow-up in these patients. For patients with a rare hemostasis pathology, it is important to have specialized follow-up by a hemostasis physician who can adapt the treatments of the pathology to all life events (surgery, pregnancy, long travel). Referring patients with menorrhagia to the rare gynecological diseases center allows for an assessment, to propose a suitable hormonal treatment for the patients, and to direct them to a hemostasis physician to set up joint follow-up. For patients followed for a chronic rare pathology, the specialized skills of the rare gynecological diseases center allow addressing gynecological issues while adapting to the specifics of the underlying rare chronic pathology. For example, in front of a young patient followed for a heart condition treated with anticoagulants, the gynecology consultation allows quantifying the bleedings and proposing a hormonal treatment adapted to her pathology and treatments. In front of a patient treated for epilepsy whose seizures increase during menstruation, achieving therapeutic amenorrhea could reduce catamenial seizures. The specialized gynecology consultation allows proposing a suitable treatment while taking into account the impact of enzyme-inducing antiepileptic treatments and reminding that contraceptive efficacy is not assured with hormonal treatments taken orally or subcutaneously. Among patients with intellectual disabilities, menstrual suppression may be used to facilitate care [11].

The referral center for rare gynecological diseases at Necker is part of the Department of Endocrinology, Diabetology, and Pediatric Gynecology. It consists of 3 gynecologists specialized in pediatric gynecology and 2 interns who work in association with pediatric endocrinologists and diabetologists. The Necker referral center for rare gynecological diseases also includes a pediatric surgeon specialized in gynecology and a psychologist. November 2018 was deliberately chosen as a start, since it corresponds to the date on which the pediatric gynecology team was permanently composed of 3 gynecologists and 2 interns to get as close as possible to the current situation. Gynecological evaluations can be done either in a gynecology consultation or in the different Necker Departments, both in the pediatric emergency room and during hospitalization. Weekly meetings are organized between the physicians of the service to discuss complex cases. National meetings via videoconference with other referral centers for rare gynecological diseases in France are organized every 2 months to share practices and discuss the most complex cases. The pediatric gynecologists in Necker hospital also provide consultations for young patients without rare chronic diseases referred by healthcare providers for gynecological issues.

In the present study, it has not been feasible to precisely collect the type of referring physician for patients seen for consultation for patients seen for uterovaginal malformations, rare breast diseases, or hereditary hemostasis diseases. Patients were either referred for consultation by pediatricians or gynecologists or seen in the pediatric emergency room or hospitalized at Necker hospital at the request of other specialists. Patients in the subgroup with gynecological repercussions of rare chronic diseases had been referred by the rare disease center following their main disease. Our presence in the Department of Endocrinology, Diabetology, and Pediatric Gynecology explains the large proportion of patients referred by the center for Rare Endocrine Diseases affecting growth and development.

On the basis of the literature analysis, the present work appears to be the first providing information on the age at and reason for gynecological visits in patients with rare chronic diseases of childhood and adolescence. The large number of patients with gynecological issues related to rare chronic diseases is a strength of our study.

One of the limitations of our study is the retrospective design. However, data for patients seen at the recruiting center are entered into the BaMaRa database according to a standardized protocol. Nonetheless, several points of interest are not recorded, such as age at menarche, treatments offered for the gynecological condition, treatments taken for an underlying rare chronic disease, and HPV immunization status. Thus, the range of data available for analysis was limited.

This study focused solely on patients with a rare chronic disease. Due to the small subgroup samples, which themselves consist of various heterogeneous pathologies, it is challenging to compare patients and derive global recommendations. Unfortunately, a systematic database for patients without a rare chronic disease does not exist, so comparing patients seen in pediatric gynecology consultations for a rare chronic disease with those without has not been possible. Data on the percentage of rare disease diagnoses based on the reasons for initial consultations in pediatric gynecology are lacking. Further studies on all patients seen in pediatric gynecology consultations at Necker Hospital could be interesting to supplement this data.

## Conclusions

Pediatric and adolescent gynecology is a developing field crucial to protecting the health, well-being, and sexual and reproductive future of young females. Centers offering expertise in rare gynecological conditions and in the gynecological repercussions of rare chronic pediatric illnesses are needed. Repercussions of rare chronic diseases managed at rare-disease referral centers were by far the leading reason for seeking gynecological expertise in rare diseases. The underlying disease and its treatments interact with the gynecological manifestations and their treatment, requiring close collaboration among all specialists caring for each patient.

It is essential to better sensitize pediatricians to pediatric gynecology. A dedicated time in consultation could be devoted to general gynecological issues: the age of the first menstrual cycles, the regularity of the cycles, the duration and abundance of the periods, the presence or absence of dysmenorrhea, and the necessity or not of contraception to be adapted according to the age of the young girls. Systematic questioning would allow highlighting issues sometimes underestimated by young girls and their families. A specialized pediatric gynecology consultation could then be proposed depending on the request. Every young girl followed for a rare disease should have a gynecological consultation to address several themes: helping them enter into their sexuality, and adult life, discussing HPV vaccination, talking about their future fertility, and planning a possible future pregnancy.

It is difficult to issue global recommendations given the diversity of chronic diseases and the heterogeneity of the subgroups. It is essential to encourage pediatricians to screen for the gynecological issues of their patients. The existence of rare disease referral centers allows the establishment of dedicated circuits for rare diseases and tends to propose a comprehensive management appropriate to the specificities of each patient. To date, there is no systematized circuit by pathology for referral to gynecology consultation at Necker Hospital. It would be interesting to develop these circuits by pathology with specialists.

## Data Availability

The data that support the findings of this study are not publicly available, due to legislation on patient-data confidentiality. However, the data are available from the authors upon reasonable request and ethics committee permission.

## Abbreviation

HPV: Human papillomavirus
